# A New Prognostic Signature Constructed with Necroptosis-Related lncRNA in Bladder Cancer

**DOI:** 10.1155/2022/5643496

**Published:** 2022-11-15

**Authors:** Zuhu Yu, Bin Lu, Hong Gao, Rongfang Liang

**Affiliations:** Department of Urology, University of Chinese Academy of Sciences Shenzhen Hospital, Guangming, Shenzhen, China

## Abstract

**Background:**

Bladder cancer (BC) accounts for the most common urologic malignancy, leading to a heavy social burden over the world. We aim to search for a novel prognostic biomarker with necroptosis-related lncRNAs of bladder cancer in this study.

**Methods:**

We download the RNA-sequencing data and corresponding clinical information of BC patients from TCGA. We performed Pearson correlation analysis to identify necroptosis-related lncRNAs (NRlncRNAs). Then, we used univariate Cox regression, Lasso Cox analysis, and multivariate Cox regression to construct the optimal prognostic model. Next, we used Kaplan–Meier curves, Cox regression, receiver operating characteristic (ROC) curves, nomogram, and stratified survival analysis to evaluate the capacity of the prognostic signature. Furthermore, gene set enrichments in the signature and the correlation between prognostic signature and necroptosis genes, tumor microenvironment, immune infiltration, and immune checkpoints of BC were also explored.

**Results:**

A 7-NRlncRNAs signature comprising FKBP14-AS1, AL731567.1, LINC02178, AC011503.2, LINC02195, AC068196.1, and AL136084.2 was constructed to predict the prognosis of BC in this research. Cox regression analysis showed that the signature could be an independent prognostic factor for BC patients (*P* < 0.001). Compared to other clinicopathological characteristics, this signature displayed a better capacity of prediction with the area under the curve (AUC) of 0.745. Stratified analysis using various clinical variables demonstrated that the prognostic signature has good clinical fitness. GSEA showed that focal adhesion and the WNT signaling pathway were enriched in the high-risk group. Immune infiltration analysis indicated that the signature was significantly inversely correlated with infiltration of CD8^+^ T cells and CD4^+^ T cells while positively correlated with macrophages and cancer associated fibroblasts. Immune checkpoint analysis revealed that the expressions of protective factors were significantly lower in the high-risk group, while expressions of cancer promotors were significantly higher in this group. The gene expression analysis displayed that necroptosis genes such as FADD, FAS, MYC, STAT3, PLK1, LEF1, EGFR, RIPK3, CASP8, BRAF, ID1, GATA3, MYCN, CD40, and TNFRSF21 were significantly different between the two groups.

**Conclusions:**

The 7-NRlncRNAs signature can predict the overall survival of BC and may provide help for the individualized treatment of BC patients.

## 1. Introduction

As the most common malignance in the urinary system, bladder cancer (BC) leads to a heavy social burden with over 200000 related deaths worldwide annually [1]. Many risk factors such as smoking, chronic infection or irritation, and occupational exposure to carcinogenic chemicals have been found associated with occurrence of BC [2]. However, the pathogenesis of BC is still unclear. BC patients with similar histology or pathological stage may have completely different prognosis. Patients with nonmuscle-invasive bladder cancer (NMIBC) have a 90% 5-year survival rate, but 50–70% of these patients will relapse, and 10–20% of them will develop muscle-invasive bladder cancer (MIBC), of which the 5-year survival rate is less than 50% [3]. Thus, to seek for a reliable and specific biomarker for the prognosis of BC is urgently needed in the clinical therapy.

Necroptosis is a novel form of programmed cell death mediated by RIPK1 (receptor-interacting protein kinase 1), RIPK3, and MLKL (mixed lineage kinase domain-like pseudokinase), presenting a mechanistic resemblance to apoptosis and a morphological resemblance to necrosis [4, 5]. Necroptosis has been confirmed to play critical roles in infection diseases and noninflammatory diseases, including oncogenesis, metastasis, and immune escape of cancer [6, 7]. Interestingly, necroptosis can hamper tumor progression in some tumors when apoptosis failed to be induced on the one hand and provoke the inflammatory responses and promote cancer immunosuppression in some cancers on the other hand [8]. These dual effects on cancers hint that targeting necroptosis would be a new strategy for the remedy of cancers, especially immunotherapy of cancers such as BC.

Long noncoding RNA (LncRNA) is a type of nonprotein-coding RNA with a length of more than 200 nucleotides existing in the nucleus or cytoplasm. Although lncRNA lacks meaningful open reading frame, it plays crucial roles in the proliferation, differentiation, and apoptosis of cells, including the oncogenesis and progression of cancers [9]. For example, LINC01614 promotes the proliferation, migration, and invasion of bladder cancer cells through the miR-217/RUNX2/Wnt/*β*-catenin axis [10]. LncRNA SLC16A1-AS1 induces metabolic reprogramming of BC cells towards favoring invasiveness by acting as a target and coactivator of E2F1 in bladder cancer [11]. Up to now, there have been few research works of lncRNAs related to necroptosis and necroptosis-related lncRNAs (NRlncRNAs) in BC that have not been studied.

In our research, we identified NRlncRNAs in BC using transcript data from TCGA and constructed a prognostic signature with differentially expressed NRlncRNAs between bladder cancer tissues and normal tissues. Then, we analyzed and evaluated its capacity of predicting overall survival of BC by different methods. Furthermore, we explored the relationships between this signature and immune infiltration, immune checkpoint, and anticancer drug sensitivity.

## 2. Materials and Methods

### 2.1. Data Acquisition

The RNA-sequencing data and relevant clinical information of bladder cancer tissues and normal tissues were downloaded from The Cancer Genome Atlas (TCGA, https://portal.gdc.cancer.gov/) in March 2022. The RNA-sequencing data were normalized to fragments per kilobase million (FPKM) format and preprocessed with Perl language (Version strawberry-perl-5.32.1.1; https://www.perl.org/) to obtain gene expression matrix of BC and normal tissues. Patients with missing overall survival values (OS) or short survival (OS < 30 days) in this study were excluded to reduce statistic bias.

### 2.2. Identification of Necroptosis-Related lncRNAs

We obtained a list of 67 necroptosis genes from previously mentioned literature in PubMed [12]. The correlation between necroptosis genes and lncRNAs in BC was analyzed with Pearson correlation analysis. Then, NRlncRNAs were identified with the standard of |Coefficient| > 0.5 and *P* < 0.001. Subsequently, we screened out the differentially expressed NRlncRNAs in BC and normal tissues with the standard of |Log 2 fold change| > 1 and *P* < 0.05 using limma R package. The network of mutual regulation between these lncRNAs and target genes was visualized with “igraph” package in R language.

### 2.3. Construction and Verification of the Prognostic Signature

All enrolled BC patients were randomly divided into the training set and testing set at the ratio of 1 : 1. In the training set, univariate Cox regression analysis was performed to identify NRlncRNA related to prognosis of BC patients (*P* < 0.05). Lasso Cox analysis was applied to determine the optimal NRlncRNAs associated with BC patients' prognoses via the glmnet R package. To prevent overfitting, 10-foldcross-validation and *P* < 0.05 were set in the processing. Subsequently, multivariate Cox regression analysis was used to construct a predictive model with the optimal NRlncRNAs [13]. Then, the risk score of each BC patients was calculated according to the expression levels of the NRlncRNAs and corresponding regression coefficients with the following formula: risk score = ∑_*k*=1_^*n*^coef(lncRNA^*k*^) × exp(lncRNA^*k*^). Coef (lncRNA) represents the coefficient of each lncRNA in the model, while exp (lncRNA) represents the expression levels of the lncRNA. Finally, all patients were divided into the high-risk group or low-risk group according to the median risk score. Using the “survival” package in R, Kaplan–Meier curves were plotted, and the log-rank test was performed to compare whether there was a difference of survival between the high-risk group and the low-risk group. The testing set and entire set were used to validate the prognostic model.

### 2.4. Assessment of the Prognostic Signature

Univariate and multivariate Cox regression analyses were used to examine where the risk score of the model was an independent prognostic factor for BC patients. Receiver operating characteristic (ROC) curves of the prognostic signature and clinical characteristics were plotted, and the area under the curve (AUC) was calculated with “survival,” “survminer” and “timerROC” packages in R. A nomogram was generated to predict 1-year, 3-year, and 5-year overall survival of BC patients by combining risk score and age, gender, clinical stage, T stage, N stage, M stage, and grade classification. Calibration curves were plotted to evaluate whether the nomogram was consistent with the actual situation. In this process, “survival,” “regplot,” and “rms” packages in R were used. In addition, stratified survival analysis according to different clinicopathological characteristics was performed to further evaluate the capacity of the prognostic signature.

### 2.5. Pathway Analysis with GESA

To identify significantly enriched pathways between the low-risk group and the high-risk group, we administered Kyoto Encyclopedia of Genes and Genomes (KEGG) pathway enrichment analysis with GSEA software (version 4.2.3).

### 2.6. Exploration of Immune Infiltration, TME, Immune Checkpoints, and Necroptosis Genes

The immune infiltration values of BC patients from TCGA were calculated based on algorithms including XCELL, TIMER, QUANTISEQ, MCPCOUNTER, EPIC, CIBERSORT-ABS, and CIBERSORT. The correlations between immune cells and risk scores were evaluated using Spearman correlation analysis and visualized with a bubble chart. In this process, the Wilcoxon signed-rank test and R packages including “limma,” “scales,” “ggplot2,” “ggtext,” “tidyverse,” and “ggpubr” were used. The tumor microenvironment (TME), infiltrated immune cells, immune checkpoint activation, and necroptosis gene expression between two risk groups were analyzed with the ESTIMATE algorithm, single sample gene set enrichment analysis (ssGSEA), and “ggpubr” package in R language, respectively.

### 2.7. Clinical Significance of the Signature in Drug Therapy

We used the “pRRpphetic” package in R language to assess the differences of responses to drug therapy between the low-risk group and the high-risk group. The response to anticancer drugs was determined by half-maximal inhibitory concentration (IC50) of patients in Genomics of Drug Sensitivity in Cancer (GDSC) (https://www.cancerrxgene.org/).

## 3. Results

### 3.1. Necroptosis-Related lncRNAs in BC

**Figure 1:**
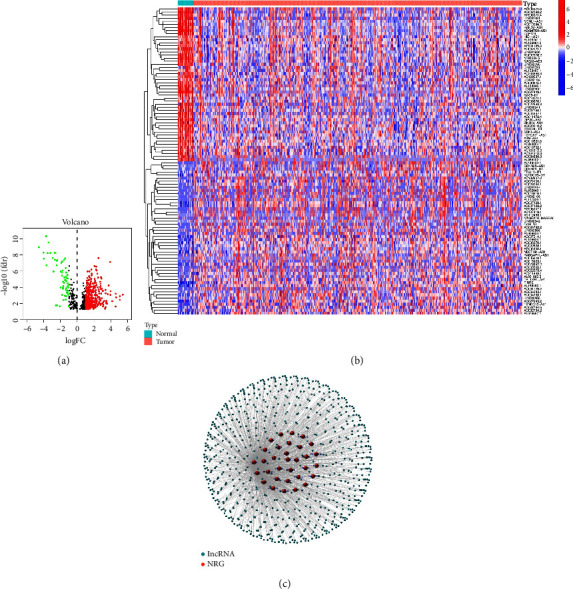
Identification of necroptosis-related lncRNA in BC. (a) Volcano plots of 440 differentially expressed NRlncRNAs in BC were identified (|Log 2 fold change| > 1, *P* < 0.05). The red spot represents lncRNA up-regulated in BC tissues, and the green spot represents lncRNA down-regulated in BC tissues. (b) The heatmap of the 100 lncRNAs with most significance. (c) The network between lncRNAs and target genes (|Coefficient| > 0.5, *P* < 0.001).

We obtained the transcriptome RNA-seq and clinical data of 411 BC tissues and 19 normal tissues from TCGA database. By Pearson correlation analysis and expression analysis, 440 NRlncRNAs were identified differentially expressed in BC tissues and normal tissues (|Log 2 fold change| > 1 and *P* < 0.05), including 59 down-regulated lncRNAs and 381 up-regulated lncRNAs, as shown in Figure 1(a). The heatmap of the 100 lncRNAs with most significance (50 up-regulated lncRNAs and 50 down-regulated lncRNAs) is shown in Figure 1(b). The regulation network between these lncRNAs and target genes is shown in Figure 1(c).

### 3.2. Construction of the Prognostic Signature

**Figure 2:**
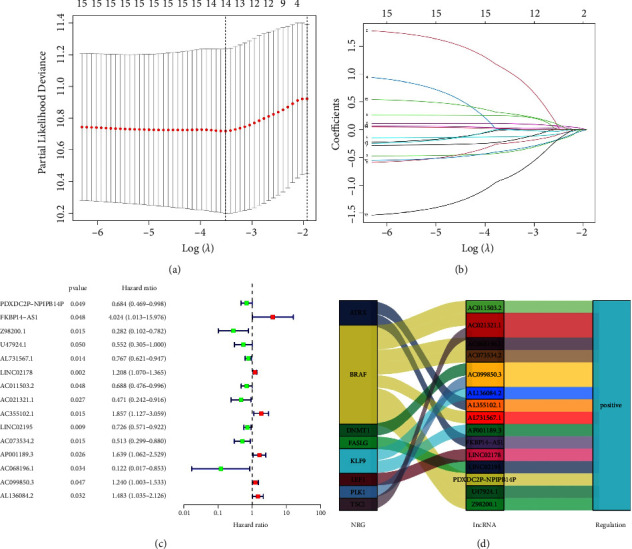
Construction of the prognostic signature using NRlncRNAs. (a) The Lasso coefficient profile of 7 NRlncRNAs in the signature. (b) The 10-foldcross-validation for variable selection in the Lasso model. (c) The 15 NRlncRNAs related to prognosis of BC were obtained using univariate Cox regression analysis (*P* < 0.05). (d) Sankey diagram showed positive regulation between necroptosis genes and 15 NRlncRNAs.

Using univariate Cox regression analysis and Lasso Cox regression analysis, we obtained 15 lncRNAs correlated to the overall survival of BC patients when the first-rank value of Log(*λ*) was the minimum likelihood of deviance (Figures 2(a) and 2(b)). The *P* values and hazard ratio of these lncRNAs are shown in Figure 2(c). The Sankey diagram showed positive regulation between necroptosis genes and all lncRNAs obtained (Figure 2(d)). Then, in the training set, we constructed the optimal prognostic model with 7 lncRNAs using multivariate Cox regression analysis. The risk score of each BC patient is calculated with the formula: risk score = (1.91355×FKBP14-AS1^exp^) + (−0.20286×AL731567.1^exp^) + (0.13955×LINC02178^exp^) + (−0.40705×AC011503.2^exp^) + (−0.51803×LINC02195^exp^) + (−1.87247×AC068196.1^exp^) + (0.44086×AL136084.2^exp^).

**Figure 3:**
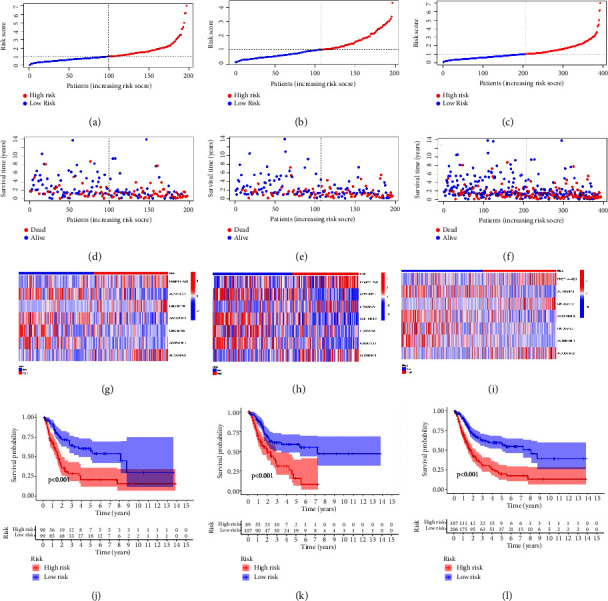
Prognostic value of the signature in the training, test, and entire set. (a–c) The distributions of the risk score in the training, test, and entire set, respectively. (d–f) The survival time and survival status of the patients in training, test, and entire set, respectively. (g–i) Heatmaps of 7 NRlncRNAs expression in BC in the training, test, and entire set, respectively. (j–l) Kaplan–Meier curves showed the overall survival between the low-risk group and the high-risk group in the training, test, and entire set, respectively (*P* < 0.001).

Based on the median value of risk score, each patient was sorted into the low-risk group or high-risk group. The distribution of the risk score and survival status of the training set, test set, and entire set are shown in Figures 3(a)–3(f), suggesting that deaths increased as the risk score elevated. Heatmaps of Figures 3(g)–3(i) showed that FKBP14-AS1, LINC02178, and AL136084.2 were up-regulated in the high-risk group, while AL731567.1, AC011503.2, LINC02195, and AC068196.1 were up-regulated in the low-risk group. Kaplan–Meier curves of various sets indicated that patients with a higher risk score had worse overall survival compared to patients with a lower risk score (Figures 3(j)–3(l), *P* < 0.001).

### 3.3. Assessment of the Prognostic Signature

**Figure 4:**
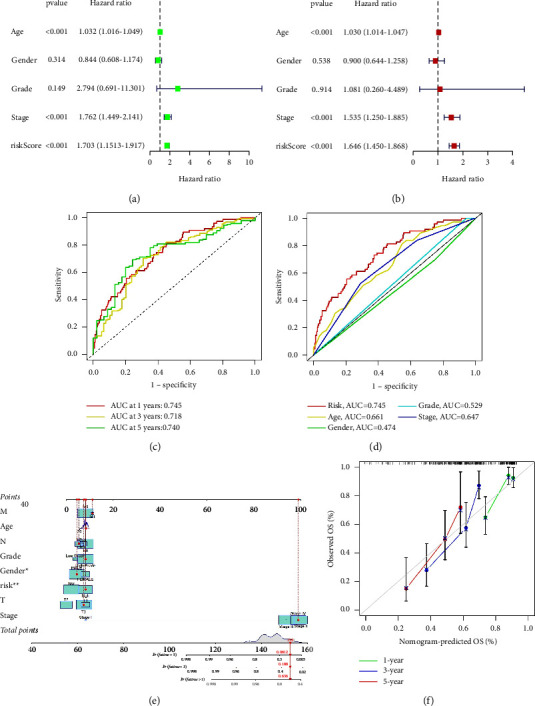
Assessment of the signature constructed with 7-NRlncRNAs. (a) Univariate Cox regression analysis of clinical characteristics and risk scores. (b) Multivariate Cox regression analysis of clinical characteristics and risk scores. (c) The AUC of 1-year, 3-year, and 5-year survival of risk scores in the entire set. (d) ROC analysis of age, gender, grade, stage, and risk scores in the entire set. (e) A nomogram combining clinicopathological characteristics and risk scores could predict the 1-year, 3-year, and 5-year survival rate of BC patients. (f) The calibration curves showed good consistence between predicted survival and practical outcomes.

Univariate Cox regression analysis revealed that the hazard ratio of age, stage, and risk score were 1.032 (95% CI = 1.016–1.049, *P* < 0.001), 1.762 (95% CI = 1.449–2.141, *P* < 0.001), and 1.703 (95% CI = 1.513–1.917, *P* < 0.001), respectively. Multivariate Cox regression analysis showed that the hazard ratio of age, stage, and the risk score were 1.030 (95% CI = 1.014–1.047, *P* < 0.001), 1.535 (95% CI = 1.250–1.885, *P* < 0.001), and 1.646 (95% CI = 1.450–1.868, *P* < 0.001), respectively. The results of Cox regression analysis suggested that the risk score is an independent prognostic factor for BC patients (Figures 4(a) and 4(b)). We drew ROC curves and calculated the AUC of the model to assess the sensitivity and specificity of the prognostic model. The AUC of 1-year, 3-year, and 5-year survival of the risk score were 0.745, 0.718, and 0.740, respectively, demonstrating that this signature has a favorable predictive ability (Figure 4(c)). Compared to other clinicopathological characteristics such as age (AUC = 0.661), grade (AUC = 0.474), and clinical stage (AUC = 0.647), the risk score (AUC = 0.745) displayed a better capacity of prediction (Figure 4(d)).

**Figure 5:**
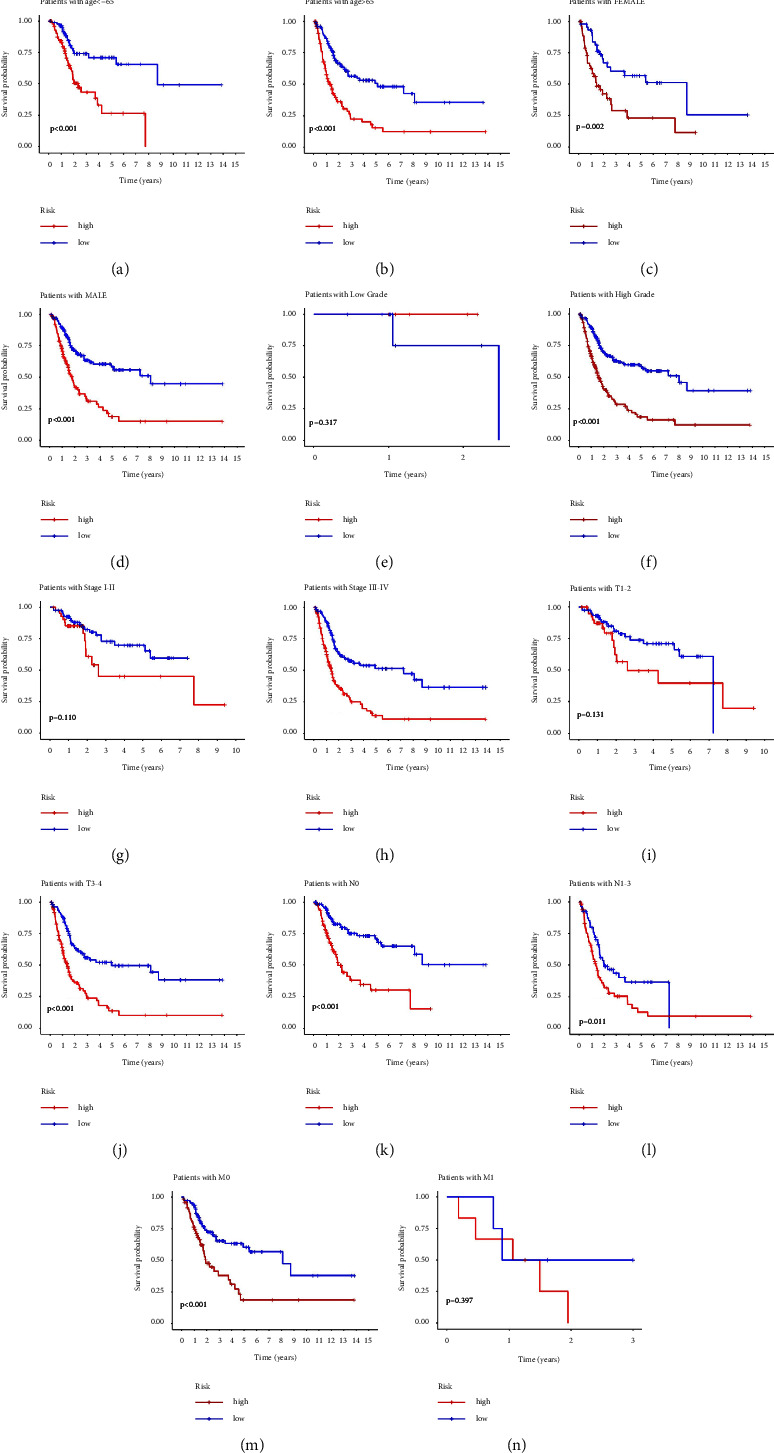
Kaplan–Meier curves of two risk groups stratified by age (a, b), gender (c, d), grade (e, f), clinical stage (g, h), T stage (i, j), N stage (k, l), and M stage (m, n).

In addition, a nomogram comprising clinicopathological features and risk scores was set up to predict the 1-year, 3-year, and 5-year survival rate of BC patients (Figure 4(e)). It is interesting that gender showed significant difference in calculation of points in this nomogram (^*∗*^*P* < 0.05), while gender was not an independent factor of prognosis. This difference may be affected by the significant gender differences of BC patients in TCGA database and different incidence of BC between men and women. The calibration curves showed that the nomogram has good consistence with practical outcomes (Figure 4(f)). To further assess the clinical applicability of the prognostic signature, we carried out stratified analysis using various clinical variables. As shown in Figures 5(a)–5(n), in the subsets of age >65 and age <65, females and males, high grade, stages III-IV, T3-4, M0, N0, and N1-3, patients with a high-risk score had worse prognosis than those with a low-risk score (*P* < 0.05), demonstrating that the prognostic signature has good clinical fitness.

### 3.4. Pathway Enrichment Analysis

**Figure 6:**
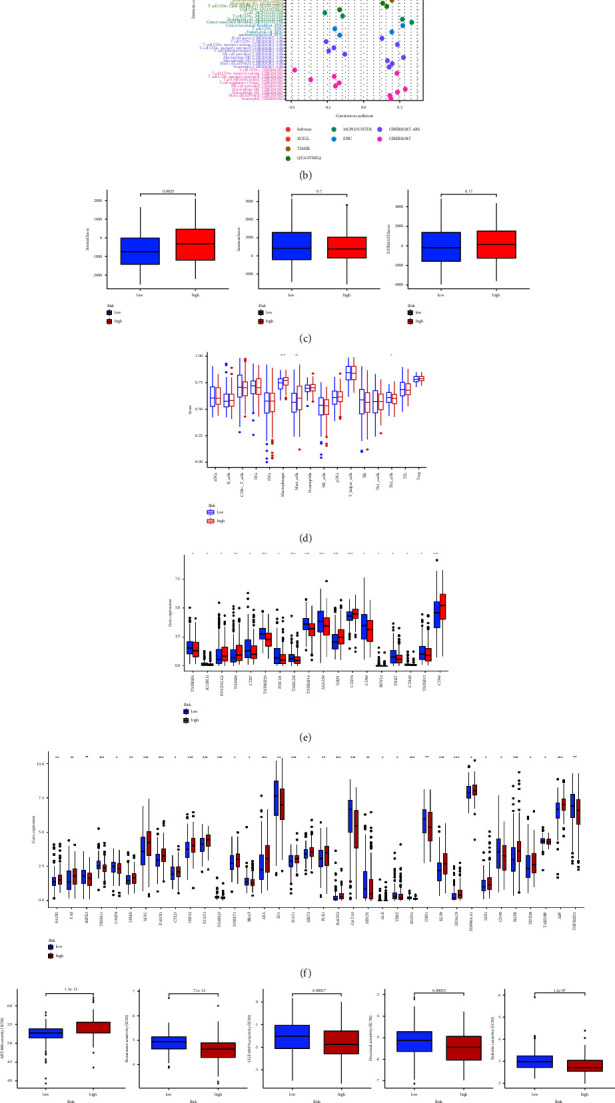
Exploration of tumor immune factors and drug therapy of BC. (a) The GSEA of top 10 pathways significantly enriched in the high-risk group (*P* < 0.001, FDR < 0.25, |NES| > 2.0). (b) The bubble chart of immune cells and risk groups. (c) Comparison of TME between the low-risk group and the high-risk group. (d) Immune cells analysis between the two groups by ssGSEA scores. (e) The comparison of immune checkpoint activation between the two risk groups. (f) The expression of necroptosis genes in two groups. (g) The differences of 10 common anticancer drug sensitivity between the two groups. ^*∗*^*P* < 0.05, ^*∗∗*^*P* < 0.01, and ^*∗∗∗*^*P* < 0.001.

We performed KEGG pathway analysis with GSEA software to analyze the pathway enrichment between the low-risk group and the high-risk group. As illustrated in Figure 6(a), the top 10 pathways enriched in the high-risk group were focal adhesion, melanoma, prostate cancer, regulation of actin cytoskeleton, WNT signaling pathway, renal cell carcinoma, endometrial cancer, pathways in cancer, gap junction, and cardiomyopathy (*P* < 0.001, FDR < 0.25, |NES| > 2.0). The result revealed that pathway enrichment in the high-risk group was significantly correlated with tumorigenesis and tumor invasion. In the low-risk group, no significant enrichment of pathways was observed (FDR > 0.25).

### 3.5. Exploration of Immune Infiltration, TME, Immune Checkpoints, and Necroptosis Genes

We used Spearman correlation analysis to evaluate the correlation between 22 types of common immune cells and risk scores, and the result is illustrated as a bubble chart in Figure 6(b). The result revealed that the risk score was significantly negatively correlated with CD8^+^ T cells, plasma B cells, CD4^+^ T cells, and T cells (|cor| > 0.2, *P* < 0.001), while it was positively correlated with macrophages, cancer associated fibroblasts, and M0 macrophages endothelial cells (|cor| > 0.2, *P* < 0.001). By the ESTIMATE algorithm, we found higher stromal infiltration in the high-risk group (Figure 6(c), *P*=0.0025). The result of ssGSEA analysis also revealed higher concentrations of macrophages and mast cells and lower concentration of Th2 cells in the high-risk group, indicating that there is a different TME between the two groups (Figure 6(d)). Comparison of immune checkpoint activation between two risk groups displayed that the expressions of TNFRSF4, ICOSLG, CD27, TNFRSF25, PDCD1, TMIGD2, TNFRSF14, LGALS9, CD40, BTNL2, TIGIT, CD160, and TNFRSF15 were significantly lower in the high-risk group, while expressions of PDCD1LG2, TNFSF9, NRP1, CD276, and CD44 were significantly higher in this group (Figure 6(e)). The expression of necroptosis gene analysis showed that genes such as FADD, FAS, MYC, STAT3, PLK1, LEF1, and EGFR were significantly overexpressed in the high-risk group, while RIPK3, CASP8, BRAF, ID1, GATA3, MYCN, CD40, and TNFRSF21 were down-regulated in the high-risk group (Figure 6(f)).

### 3.6. Differences of Anticancer Drug Sensitivity between Two Subsets

There were significant differences in the responses to anticancer drugs between the high-risk group and the low-risk group (Figure 6(g)). Patients in the low-risk group were more sensitive to ABT.888 (veliparib), gefitinib, methotrexate, and Nutlin-3a, while patients in the high-risk group were more sensitive to bexarotene, CGP.60474, docetaxe, embelin, imatinib, and pazopanib. This result may provide a reference for individualized drug therapy for BC patients.

## 4. Discussion

A number of research works have explored the signatures of prognosis and classification of BC with SUMOylation, ferroptosis, immune infiltration-related lncRNA, and toll-like receptor 4 or pyroptosis-associated lncRNA [14, 15]. However, there were only a few studies concentrated on the effect of necroptosis in BC at present. For example, researchers have found that the PKM2 (pyruvate kinase M2) inhibitor shikonin could kill the T24 cisplatin resistant cells by inducing necroptosis rather than apoptosis [16]. Another study in vitro revealed that ABT-737, a BCL-2 family inhibitor, could restrain the proliferation and invasion of bladder cancer cells by inducing necroptosis [17]. Up to now, there is no study of the NRlncRNAs in BC. In this study, we are the first to identify the lncRNAs associated with necroptosis in BC and to construct a prognostic signature of NRlncRNAs. Further analysis demonstrated that the risk score of this signature is an independent prognostic factor and presents a favorable predictive ability of the outcome of the BC patients.

The prognostic signature of BC comprises 7 NRlncRNAs, namely, FKBP14-AS1, LINC02178, AL136084.2, AL731567.1, AC011503.2, LINC02195, and AC068196.1. Among these lncRNAs, LINC02195 has been illustrated as a regulator of MHC I (major histocompatibility complex class I) molecules, which plays a crucial role in the immunosurveillance in head and neck squamous cell carcinoma [18]. High expression of LINC02195 is positively correlated with an increased number of CD8^+^ and CD4^+^ T cells in the tumor microenvironment, proffering a favorable prognosis in patients with head and neck squamous cell carcinoma. Interestingly, we discovered that LINC02195 was up-regulated in the low-risk group and correlated to a favorable prognosis of BC patients in this research, suggesting that LINC02195 may act as a tumor suppressor and plays an important role in the immunity of BC. The regulation network revealed that LINC02195 positively regulates FASLG, which together with FAS initiates cell death and prevents tumor progression [19]. LINC02178 was identified as an autophagy-related lncRNA and negatively correlated to the prognosis of BC in a previous study [20]. AC011503.2 was identified as a glycolysis-related lncRNA and a protective factor of the prognosis of BC [21]. Our research also revealed that LINC02178 and AC011503.2 were up-regulated and down-regulated in the high-risk group, indicating that these lncRNAs may play important roles in the cell death and metabolism of BC. Furthermore, AL731567.1, AC011503.2, and AC068196.1 were found down-regulated in the high-risk group and positively regulated in BARF in this study. The mutations of BARF have been demonstrated to promote many cancers such as melanoma, lung cancer, and colorectal cancer [22]. The result also indicated that these lncRNAs may be involved in the regulation network of bladder cancer.

The result of GSEA showed that the WNT signaling pathway, focal adhesion, and gap junction are enriched in the high-risk group. Numerous research works have demonstrated that aberrant activation of the WNT signaling pathway plays important roles in the pathological process of many cancer types, including BC [23, 24]. Target genes of the WNT pathway induce the epithelial-mesenchymal transition (EMT) process of cancers by involving in cell adhesion, leading to conversion of nontumorigenic cells into cancer stem cells. Besides, many genes in the WNT pathway were found associated with interferon signaling, proffering that this pathway participates in the interferon-mediated immune responses of cancer cells. Therefore, the KEGG enrichment analysis also confirms that patients in the high-risk group have high malignance and worse prognosis.

Immune infiltration analysis indicated that the risk score was significantly inversely correlated with infiltration of CD8^+^ T cells and CD4^+^ T cells and positively correlated with macrophages and cancer-associated fibroblasts. Prior research works have proven that CD8^+^ T cells and CD4^+^ T cells are the crucial defenders in the antitumor immunity [25–27]. The dysfunction and exhaustion of cytotoxic T cells contribute to the immune related tolerance and immunosuppression during the progression of cancers. On the other hand, high infiltration of macrophages and cancer-associated fibroblasts has been evidenced to have been associated with the tumor progression and poor outcomes of patients [28–31]. The further analysis of immune checkpoints in this study revealed that the expressions of protective factors such as TNFRSF4, CD27, TNFRSF25, PDCD1, TNFRSF14, CD40, and TIGIT were significantly lower in the high-risk group, while expressions of cancer promotors such as PDCD1LG2, TNFSF9, NRP1, CD276, and CD44 were significantly higher in this group, also suggesting that the high-risk group has poor overall survival [32–35]. In addition, we discovered that the expression of necroptosis genes such as RIPK3, FADD, FAS, MYC, STAT3, PLK1, EGFR, CASP8, BRAF, ID1, GATA3, and CD40 were significantly different in the high-risk and low-risk groups. Abundant research works have confirmed that these genes play crucial roles in the oncogenesis, progression, and drug resistance of cancers [36–39]. The expression of PLK1, GATA3, and CD40 were even related to the prognosis of bladder cancer [40–42]. This result indicated that necroptosis may partly lead to the differences in the survival of the high-risk and low-risk group.

The present study also has certain limitations. Firstly, only TCGA database was used for internal validation in this research, and more datasets are needed to further verify the capacity of the signature. Secondly, the prognostic signature was established based on the transcriptome RNA-seq and clinical data of BC patients form TCGA, and expression of lncRNAs in this signature needs more verification with clinical specimens from different centers. In addition, the functions and regulatory pathways of the NRlncRNAs in BC need further explorations with in vitro or in vivo experiments.

## 5. Conclusions

In this study, we constructed a prognostic signature for BC with 7 differentially expressed NRlncRNAs. This signature can predict the overall survival of BC patients and provide a reference for the individualized treatment of BC. Further explorations are needed to define the functions and pathways of NRlncRNAs in BC.

## Data Availability

The data used in this study are available in TCGA database (https://portal.gdc.cancer.gov/repository). Detailed data of the manuscript are available from the correspondence author.

## References

[1] Sung H., Ferlay J., Siegel R. L. (2021). Global cancer statistics 2020: globocan estimates of incidence and mortality worldwide for 36 cancers in 185 countries. *CA: A Cancer Journal for Clinicians*.

[2] Cumberbatch M. G., Rota M., Catto J. W., La Vecchia C. (2016). The role of tobacco smoke in bladder and kidney carcinogenesis: a comparison of exposures and meta-analysis of incidence and mortality risks. *European Urology*.

[3] Lenis A. T., Lec P. M., Chamie K., Mshs M. D. (2020). Bladder cancer: a review. *JAMA*.

[4] Christofferson D. E., Yuan J. (2010). Necroptosis as an alternative form of programmed cell death. *Current Opinion in Cell Biology*.

[5] Snyder A. G., Hubbard N. W., Messmer M. N. (2019). Intratumoral activation of the necroptotic pathway components ripk1 and ripk3 potentiates antitumor immunity. *Sci Immunol*.

[6] Khoury M. K., Gupta K., Franco S. R., Liu B. (2020). Necroptosis in the pathophysiology of disease. *American Journal Of Pathology*.

[7] Huang Y., Zou Y., Xiong Q. (2021). Development of a novel necroptosis-associated mirna risk signature to evaluate the prognosis of colon cancer patients. *Annals of Translational Medicine*.

[8] Zhu F., Zhang W., Yang T., He S. D. (2019). Complex roles of necroptosis in cancer. *Journal of Zhejiang University - Science B*.

[9] Mercer T. R., Dinger M. E., Mattick J. S. (2009). Long non-coding rnas: insights into functions. *Nature Reviews Genetics*.

[10] Wang Z., Yan H., Cheng D. (2021). Novel lncRNA LINC01614 facilitates bladder cancer proliferation, migration and invasion through the miR-217/RUNX2/Wnt/*β*-Catenin Axis. *Cancer Management and Research*.

[11] Logotheti S., Marquardt S., Gupta S. K. (2020). Lncrna-slc16a1-as1 induces metabolic reprogramming during bladder cancer progression as target and co-activator of e2f1. *Theranostics*.

[12] Zhao Z., Liu H., Zhou X. (2021). Necroptosis-related lncrnas: predicting prognosis and the distinction between the cold and hot tumors in gastric cancer. *Journal of Oncology*.

[13] Lu Y., Luo X., Wang Q. (2022). A novel necroptosis-related lncrna signature predicts the prognosis of lung adenocarcinoma. *Frontiers in Genetics*.

[14] Xia Q. D., Sun J. X., Liu C. Q. (2022). Ferroptosis patterns and tumor microenvironment infiltration characterization in bladder cancer. *Frontiers in Cell and Developmental Biology*.

[15] Lu H., Wu J., Liang L., Wang X., Cai H. (2022). Identifying a novel defined pyroptosis-associated long noncoding rna signature contributes to predicting prognosis and tumor microenvironment of bladder cancer. *Frontiers in Immunology*.

[16] Wang Y., Hao F., Nan Y. (2018). Pkm2 inhibitor shikonin overcomes the cisplatin resistance in bladder cancer by inducing necroptosis. *International Journal of Biological Sciences*.

[17] Cheng R., Liu X., Wang Z., Tang K. (2021). Abt737, a bcl2 family inhibitor, has a synergistic effect with apoptosis by inducing urothelial carcinoma cell necroptosis. *Molecular Medicine Reports*.

[18] Li H., Xiong H. G., Xiao Y. (2020). Long non-coding rna linc02195 as a regulator of mhc i molecules and favorable prognostic marker for head and neck squamous cell carcinoma. *Frontiers Oncology*.

[19] Liu Y., Wen Q. J., Yin Y. (2009). Faslg polymorphism is associated with cancer risk. *European Journal of Cancer*.

[20] Sun Z., Jing C., Xiao C., Li T. (2020). An autophagy-related long non-coding rna prognostic signature accurately predicts survival outcomes in bladder urothelial carcinoma patients. *Aging (Albany NY)*.

[21] Zheng Z., Lai C., Li W., Zhang C., Ma K., Yao Y. (2021). Identification of a novel glycolysis-related lncrna signature for predicting overall survival in patients with bladder cancer. *Frontiers in Genetics*.

[22] Dankner M., Rose A. A. N., Rajkumar S., Siegel P. M., Watson I. R. (2018). Classifying braf alterations in cancer: new rational therapeutic strategies for actionable mutations. *Oncogene*.

[23] Taciak B., Pruszynska I., Kiraga L., Bialasek M., Krol M. (2018). Wnt signaling pathway in development and cancer. *Journal of Physiology & Pharmacology*.

[24] Jimenez-Guerrero R., Belmonte-Fernandez A., Flores M. L. (2021). Wnt/*β*-Catenin signaling contributes to paclitaxel resistance in bladder cancer cells with cancer stem cell-like properties. *International Journal of Molecular Sciences*.

[25] Oh D. Y., Kwek S. S., Raju S. S. (2020). Intratumoral cd4(+) t cells mediate anti-tumor cytotoxicity in human bladder cancer. *Cell*.

[26] Farhood B., Najafi M., Mortezaee K. (2019). Cd8(+) cytotoxic t lymphocytes in cancer immunotherapy: a review. *Journal of Cellular Physiology*.

[27] Sato Y., Bolzenius J. K., Eteleeb A. M. (2018). Cd4+ t cells induce rejection of urothelial tumors after immune checkpoint blockade. *JCI Insight*.

[28] Yang F., Guo Z., He C. (2021). Cancer-associated fibroblasts promote cell proliferation and invasion via paracrine Wnt/IL1*β* signaling pathway in human bladder cancer. *Neoplasma*.

[29] Liu B., Pan S., Liu J., Kong C. (2021). Cancer-associated fibroblasts and the related runt-related transcription factor 2 (runx2) promote bladder cancer progression. *Gene*.

[30] Ruffell B., Coussens L. M. (2015). Macrophages and therapeutic resistance in cancer. *Cancer Cell*.

[31] Miyake M., Hori S., Morizawa Y. (2016). Corrigendum to “CXCL1-mediated interaction of cancer cells with tumor-associated macrophages and cancer-associated fibroblasts promotes tumor progression in human bladder cancer” [neoplasia 18 (2016) 636–646]. *Neoplasia*.

[32] Wu J., Wang Y., Yang Y. (2021). Tnfsf9 promotes metastasis of pancreatic cancer through wnt/snail signaling and m2 polarization of macrophages. *Aging (Albany NY)*.

[33] Aicher W. K., Korn M., Reitnauer L. (2021). Expression patterns of the immune checkpoint ligand cd276 in urothelial carcinoma. *BMC Urology*.

[34] Dong Y., Ma W. M., Shi Z. D. (2021). Role of nrp1 in bladder cancer pathogenesis and progression. *Frontiers Oncology*.

[35] Miao Y., Wang J., Li Q. (2020). Prognostic value and immunological role of pdcd1 gene in pan-cancer. *International Immunopharmacology*.

[36] Park H. H., Kim H. R., Park S. Y. (2021). Ripk3 activation induces trim28 derepression in cancer cells and enhances the anti-tumor microenvironment. *Molecular Cancer*.

[37] Ranjan K., Waghela B. N., Vaidya F. U., Pathak C. (2020). Cell-penetrablepeptide-conjugated fadd induces apoptosis and regulates inflammatory signaling in cancer cells. *International Journal of Molecular Sciences*.

[38] Yu H., Pardoll D., Jove R. (2009). Stats in cancer inflammation and immunity: a leading role for stat3. *Nature Reviews Cancer*.

[39] Liu Z., Sun Q., Wang X. (2017). Plk1, a potential target for cancer therapy. *Translational Oncology*.

[40] Liu B., Meng L. B., Su J. Z. (2022). Plk1 as one novel target for the poor prognosis of bladder cancer: an observational study. *Medicine (Baltimore)*.

[41] Bejrananda T., Kanjanapradit K., Saetang J., Sangkhathat S. (2021). Impact of immunohistochemistry-based subtyping of gata3, ck20, ck5/6, and ck14 expression on survival after radical cystectomy for muscle-invasive bladder cancer. *Scientific Reports*.

[42] Luo C., Lei T., Zhao M., Meng Q., Zhang M. (2020). Cd40 is positively correlated with the expression of nucleophosmin in cisplatin-resistant bladder cancer. *Journal of Oncology*.

